# Recent Advances in Targeted and Untargeted Metabolomics by NMR and MS/NMR Methods

**DOI:** 10.3390/ht7020009

**Published:** 2018-04-18

**Authors:** Kerem Bingol

**Affiliations:** Environmental Molecular Sciences Laboratory, Pacific Northwest National Laboratory, Richland, WA 99354, USA; kerem.bingol@pnnl.gov; Tel.: +1-509-371-7927

**Keywords:** targeted profiling, untargeted metabolomics, mass spectrometry, nuclear magnetic resonance spectroscopy, hybrid MS/NMR methods

## Abstract

Metabolomics has made significant progress in multiple fronts in the last 18 months. This minireview aimed to give an overview of these advancements in the light of their contribution to targeted and untargeted metabolomics. New computational approaches have emerged to overcome the manual absolute quantitation step of metabolites in one-dimensional (1D) ^1^H nuclear magnetic resonance (NMR) spectra. This provides more consistency between inter-laboratory comparisons. Integration of two-dimensional (2D) NMR metabolomics databases under a unified web server allowed for very accurate identification of the metabolites that have been catalogued in these databases. For the remaining uncatalogued and unknown metabolites, new cheminformatics approaches have been developed by combining NMR and mass spectrometry (MS). These hybrid MS/NMR approaches accelerated the identification of unknowns in untargeted studies, and now they are allowing for profiling ever larger number of metabolites in application studies.

## 1. Introduction

Omics technologies have been rapidly growing and providing holistic molecular information to comprehensively study biological systems. Through the advancements in DNA sequencing, the complete determination of the genome sequence of an organism at single nucleotide resolution has become achievable in very short time frames [1,2]. One key challenge in biology is to translate this information to determine the phenotype of an organism by combining genome, transcriptome, proteome, metabolome, structural, and physiological information. Organisms perform many of the functions through the use of small molecules and metabolites; therefore, small biological molecules, metabolites provide unique piece of information to help in shedding the light on phenotypic characteristics of genome sequence [3,4,5,6,7].

Metabolites are direct read out of also non-sequence related influences on phenotypes, such as environmental perturbations; drought, temperature, light, and atmospheric CO_2_ concentration [8,9,10]. The analysis of metabolite profiles of common biofluids, such as urine and serum, provides diagnostic and prognostic information about diseases, dietary patterns, and drug toxicity [11,12,13]. Stable isotope labeling of metabolites followed by the analysis of positional enrichments reveals activity of alternative metabolic pathways contributing to certain biochemical reactions, such as in healthy versus cancer cells [14,15,16].

Comprehensive analysis of metabolites are performed by metabolomics technologies, which provide identities and quantities of dozens to hundreds of intracellular and extracellular metabolites in biological samples by utilizing separation technologies, such as chromatography, capillary electrophoresis and ion mobility; and, detection technologies, in particular, mass spectrometry (MS) and nuclear magnetic resonance (NMR) spectroscopy [17,18]. Data acquisition of hundreds of samples has become achievable by high-throughput automated platforms, which also reduces the cost of measurement per sample.

Metabolome profiling is typically performed either by targeted or untargeted methods, which were explained briefly in the following sections. Regardless of the approach, every metabolomics strategy includes identification and quantitation of metabolites [19,20]. This minireview aimed to give an overview of the achievements in metabolomics over the past 18 months in order to improve the quantitation and identification of metabolites by NMR and hybrid MS/NMR methods [21,22]. The review covered the advancements in the light of their contribution to targeted and untargeted metabolomics.

## 2. Targeted Metabolomics

### 2.1. Quantitation of Metabolites

Targeted and semi-targeted metabolomics studies focus on accurate identification and quantitation of a defined set of metabolites in biological samples. Typically, this set of metabolites are predetermined by the scientific question at hand or the size of the metabolite library that is available in the software used for data analysis. The resulting data sets are feed into statistical data analysis tools to see how well targeted metabolites contribute to the group separation between control and phenotype of interest [23]. Correlations are further evaluated to understand underlying metabolism differences between groups [24,25].

**Figure 1:**
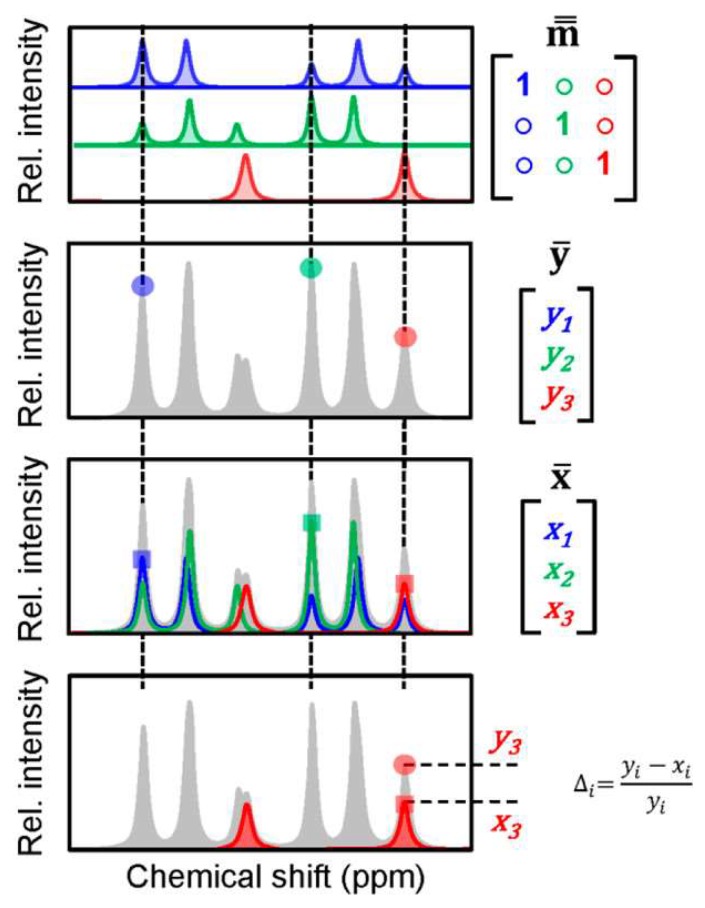
Demonstration of the Automated Quantification Algorithm (AQuA) on a hypothetical mixture of three metabolites. AQuA first applies a data reduction step to predetermine a specific signal for the quantitation of each metabolite in the mixture. Then by using a calibration spectrum for each metabolite from a library, it establishes the signal height vs. concentration relations for the selected target peaks. Finally, by using this information in the application study, AQuA determined the concentration of 67 metabolites in more than a thousand human plasma samples in less than a second, which is much faster than the previously developed quantitation approaches that were relying on all of the data points for the curve-fitting based quantitation. Adapted with permission from reference [33]. Copyright 2018 American Chemical Society. ppm: parts per million.

Metabolomics profiling by NMR has been often performed by one-dimensional (1D) ^1^H NMR experiments [26]. The advantage of 1D ^1^H NMR is its short acquisition time, which is typically less than an hour/sample and high sensitivity; it is able to detect metabolites down to single digit μM concentration [27]. When this is coupled to a manual peak fitting or a manual spike-in approach, 1D ^1^H NMR is capable to provide absolute concentration of dozens to hundred of metabolites in metabolomics samples [28]. Unfortunately, the manual analysis of 1D ^1^H NMR spectrum is labor intensive and time-consuming, especially when it is repeated for multiple samples. Speeding up this step by automation is an active research area in NMR-based metabolomics. BATMAN [29] and BAYESIL [30] are two early approaches that have been developed for automated quantitation of 1D ^1^H NMR spectra by probabilistic models, followed more recently by ASICS [31], rDolphin [32], and AQuA [33]. The workflow of the AQuA approach is demonstrated in Figure 1. All of these five approaches have been primarily developed for the quantitative profiling of aqueous metabolites, another new tool LipSpin [34] has been developed for the quantitative profiling of lipids by 1D ^1^H NMR. While these automated 1D ^1^H NMR approaches greatly speed up metabolite profiling, they still require up-front manual customization of the metabolite library to include only the metabolites that are observed in the biospecimen of interest [35]. Successful implementation of the approaches also requires careful adjustments of line-widths and peak shifts that could vary between metabolomics samples.

One-dimensional ^1^H NMR suffers from low spectral resolution along the ^1^H dimension, which limits the metabolome coverage as well as the accuracy of metabolite analysis. The recent progress focused on solving these challenges by reducing spectral overlap problems by advanced NMR methods. Spectral overlap problem can be resolved by measuring the samples at higher magnetic field strengths, which also increases the sensitivity. However, this is costly, as it requires new equipment. Alternatively, the overlap problem can be resolved by decoupling homonuclear ^1^H–^1^H scalar couplings in 1D ^1^H NMR spectra by using pure shift methods [36,37], which generate narrow peaks without multiplet splitting. However, these approaches generally cause sensitivity losses; therefore, they only allow for the profiling of high abundant metabolites. Significant improvement on resolution can be obtained by acquiring two-dimensional (2D) NMR experiments. During 2D NMR experiments, spin magnetization is transferred between neighboring nuclear spins, which are then detected in the form of cross-peaks in the final 2D spectra with much less peak overlap. Two commonly used 2D NMR experiments in metabolomics are the 2D ^13^C–^1^H heteronuclear single quantum coherence (HSQC), revealing direct one-bond correlation between the ^1^H spins with their directly attached ^13^C spins and 2D ^1^H–^1^H total correlation spectroscopy (TOCSY) experiment revealing chemical shifts of all ^1^H spins within a molecule or a spin system. The HSQC experiment is a very high resolution 2D NMR experiment due to the large spectral dispersion along ^13^C dimension and the TOCSY experiment provides connectivity information that can be reliably combined with HSQC for deconvolution of metabolites [38].

Quantitation of metabolites by 2D NMR is also an active research area in NMR-based metabolomics [39]. There are multiple approaches that have been developed primarily by using 2D ^13^C-^1^H HSQC, these methods were reviewed recently [40]. The main drawback of 2D NMR experiments is the longer acquisition time; they can easily take half a day per sample. Faster experiments are promising to speed up the data acquisition by using new pulse sequences, non-uniform sampling (NUS), and parameter optimization [40]. Sensitivity is another major problem of heteronuclear 2D NMR experiments, and progress has been achieved by combining NMR with dynamic nuclear polarization (DNP) [41,42].

### 2.2. Identification of Known Metabolites with the Use of a Database for Matching

Targeted metabolite identification is typically performed by comparing 1D ^1^H NMR spectrum of a metabolite mixture with a 1D ^1^H NMR spectral database, followed by validation of these identifications on a subset of samples by 2D NMR methods, primarily 2D HSQC and TOCSY [43,44,45]. So far, the common way of validating metabolites by these experiments have been separate and independent querying of experimental HSQC and TOCSY data against HSQC and TOCSY metabolomics databases, respectively, without taking the complementary nature of these experiments into account. To resolve this disconnectivity issue, recently, an integrated metabolite identification and validation approach, COLMARm has been introduced.

**Figure 2:**
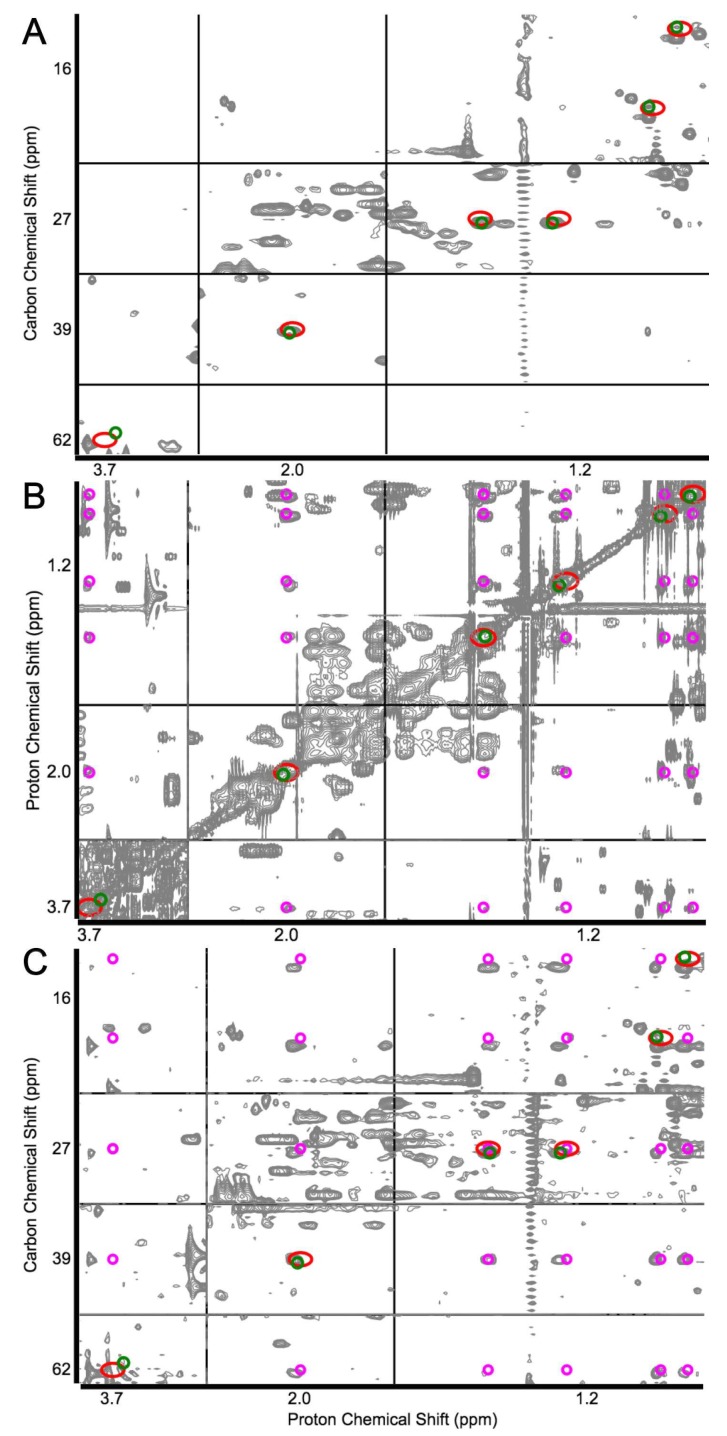
Demonstration of COLMARm web server with the identification of isoleucine in human serum by the coanalysis of (**A**) heteronuclear single quantum coherence (HSQC), (**B**) total correlation spectroscopy (TOCSY), and (**C**) HSQC–TOCSY. Green and red circles show experimental and database cross-peaks of isoleucine, respectively. Magenta circles indicate the expected isoleucine peaks from the TOCCATA database. The close agreement between green and red circles returned isoleucine as a strong candidate. This was validated by the close match between magenta peaks with the experimental TOCSY cross-peaks observed in the TOCSY and HSQC–TOCSY spectra. Adapted with permission from reference [49]. Copyright 2016 American Chemical Society.

COLMARm allows for the simultaneous analysis of 2D ^13^C–^1^H HSQC, 2D ^1^H–^1^H TOCSY, and 2D ^13^C–^1^H HSQC–TOCSY spectra of a metabolomics sample on a publicly available web server (http://spin.ccic.ohio-state.edu/index.php/colmarm/index). When the user uploads an HSQC and TOCSY-type spectra to the web server, the metabolite identification occurs at two steps. At first, the COLMARm web server queries the HSQC spectrum against COLMAR HSQC metabolomics database [46] and returns a list of candidate metabolite hits. Next, for each returned hit, the corresponding TOCSY and/or HSQC–TOCSY spectrum is generated from the COLMAR ^1^H-(^13^C) TOCCATA database [47,48] and is overlaid on the experimental TOCSY spectra [49]. Finally, depending on how well the TOCSY cross-peaks superimpose with the corresponding experimental peaks, the candidate metabolite is either manually confirmed or rejected (Figure 2). By using this strategy, the authors confidently identified 62 metabolites in human serum, 14 of these were identified in human serum for the first time by NMR, which would not be possible with high accuracy by using 1D ^1^H NMR or any other 2D NMR spectrum alone [49].

Although the NMR approaches are capable of identifying the majority of detectable metabolites in a given sample, they can fail in the discrimination of metabolites with very similar structures and chemical shifts, such as creatine versus creatine phosphate, or uridine diphosphate versus uridine triphosphate. Since often these types of metabolites possess different mass-to-charge (*m/z*) ratios, it should be possible to distinguish them by combining NMR and MS information. To accomplish this task, the NMR/MS Translator approach has been introduced [50]. The fully automated NMR/MS Translator approach first generates the metabolite candidates by querying experimental 1D or 2D NMR spectra of a sample against NMR metabolomics database. Then, for the returned candidates, it calculates the masses (*m/z*) of all their possible ions and adducts, together with their characteristic isotope distributions. Finally, it compares the expected *m/z* ratios with the experimental high resolution MS^1^ spectrum of the same sample for direct validation of the candidate metabolites that are identified by both NMR and MS [50]. The NMR/MS Translator approach has been applied to human urine, tomato extract, and *Arabidopsis thaliana* metabolome [51], by increasing the confidence of the identifications over the use of NMR or MS method alone.

## 3. Untargeted Metabolomics

Untargeted studies focus on measuring and comparing as many signals as possible across a sample set, followed by the assignment of these signal to metabolite ID’s by using metabolomics databases. Although excellent progress has been made in expanding metabolomics databases with new metabolites, a significant portion of signals detected in untargeted studies still cannot be identified through this approach due to the absence of their spectra in the databases. Untargeted studies are highly interested in the identification of unknown metabolites, especially when they are the biomarkers of a study.

Identification of unknowns is generally accepted as the bottleneck of untargeted metabolomics. Traditionally, their identification have been performed by extensive isolation of the molecule from extracts in sufficient amount for detailed analysis by MS, NMR, X-ray, circular dichroism, and other analytical techniques [52,53,54]. While this approach has been proven to be useful, complete fractionation is time-consuming. Moreover, a low yield of purification may not allow for the downstream structure elucidation process for low-abundance metabolites. Alternatively, structure elucidation can be performed in the mixture environment, such as in crude extract or the partially fractionated sample.

There are three main approaches that are proposed for unknown identification in mixtures; the first approach uses only mass spectrometry techniques. This approach compares the experimental fragmentation spectra of unknown metabolite with the predicted fragmentation of all the possible candidate isomeric structures to find the best match [55,56,57,58]. The second strategy, on the other hand, only relies on NMR, in which the experimental chemical shifts of unknown metabolites are sequentially assigned and deconvoluted by multidimensional NMR [59,60]. These assignments are further verified through comparison against their quantum NMR chemical shift predictions [61]. While these MS- and NMR-based approaches greatly facilitate the structural characterization, they have limited power, since they only rely on a single technique. Recently, several hybrid MS/NMR metabolite identification strategies have been proposed [62,63,64]. They were recently reviewed in detail [65].

**Figure 3:**
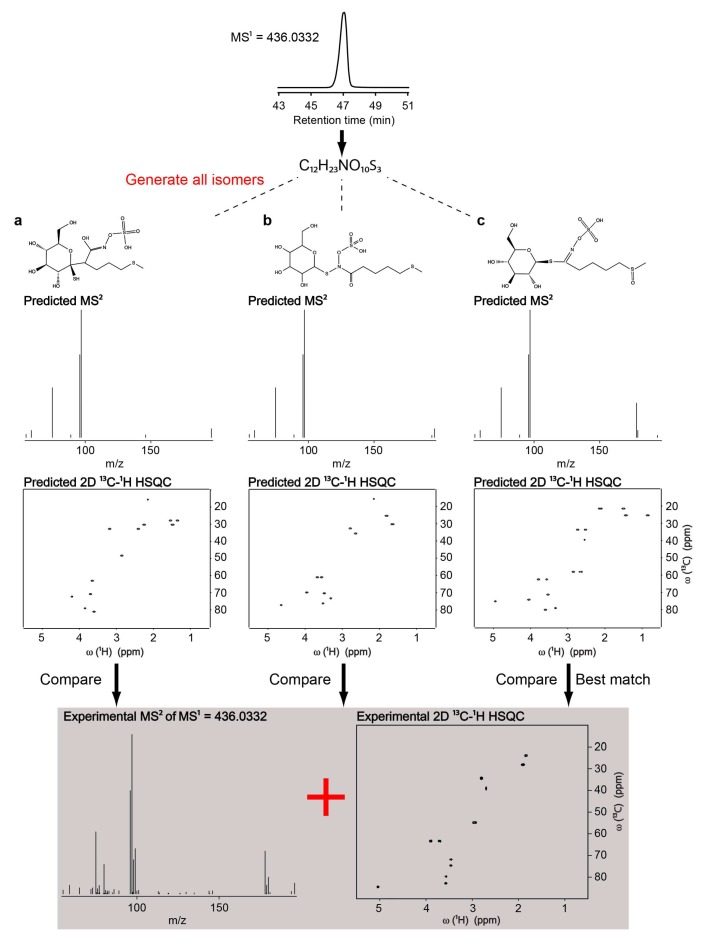
Illustration of the workflow of the Integrated Structure Elucidation (ISEL) NMR/MS^2^ approach on identification of glucoraphanin in *Arabidopsis* extract. Chemical formula of the unknown metabolite was first determined from high resolution LC-MS^1^ spectrum. Next, all of the feasible candidate structures consisted with the chemical formula were generated. MS/MS and NMR spectra of each candidate structures were predicted and compared with the experimental MS/MS and NMR spectra of the same sample and ranked according to the level of agreement to determine the best matching candidate. Adapted from reference [66].

One of the latest hybrid techniques, ISEL NMR/MS^2^ (Integrated Structure ELucidation by NMR/MS^2^) is a novel approach that combines in silico MS/MS and NMR predictions into a single analysis platform to improve the accuracy of automated unknown metabolite identification. In this approach, the unknown metabolites are first identified by determining their chemical formula from high resolution mass spectrometry coupled with liquid chromatography (LC–MS^1^). Next, all of the feasible candidate structures that are consisted with the chemical formula are generated by using a structure generator. MS/MS and NMR spectra of each candidate structures are predicted and compared with the experimental MS/MS and NMR spectra of the same sample, and finally ranked according to the level of agreement to determine the best matching candidate [66]. The authors first compared the performances of MS/MS and NMR predictions on a mixture of ten commonly known metabolites. Based on these comparisons, the NMR predictions turned out to be a more effective filter than MS/MS predictions. However, MS/MS predictions provided an orthogonal method that allowed for distinguishing between molecules that yield similar NMR chemical shifts. Thus, combining NMR and MS/MS predictions further improved the accuracy of unknown metabolite identification. As a real-world example, the authors applied the ISEL NMR/MS^2^ approach on the identification of an uncatalogued secondary metabolite in *Arabidopsis thaliana* extract, which allowed for the successful identification of glucoraphanin, a type of glucosinolate in *Arabidopsis* (Figure 3).

In the glucoraphanin example in Figure 3, which considered one unknown metabolite at a time, the NMR spectrum was paired with a single LC–MS feature by an off-line LC fractionation procedure. However, it is common in metabolomics to analyze complex mixtures that are consisting of multiple metabolites within unfractionated samples. In these cases, multiple LC–MS features and multiple deconvoluted NMR spectra are generated, without knowing which pairs correspond to the same metabolite. This increases the challenge of high-throughput metabolite identification in mixtures.

Fortunately, in cohort studies, MS and NMR features can be paired by statistical correlation. This has been recently shown in a systematic study of 26 metabolites over 200 samples. The authors showed that in a majority of cases, the highest correlated NMR and MS signals belong to the same metabolite [67]. Therefore, this approach can be utilized to inter-relate NMR and MS signals of unknowns in complex mixtures without the need for LC-fractionation.

Unfortunately for the analysis of a single sample, statistical correlation is not an option; in this case, the ISEL NMR/MS^2^ approach can still be applied. When multiple unknowns are present, the workflow is modified to consider all pairwise combinations; NMR predictions are generated for all of the structural isomers of each LC-MS feature (chemical formula), and they are all compared to every deconvoluted experimental NMR spectra of the mixture. Proof-of-principle was demonstrated on a ten-compound model mixture [66]. In 8 out of 10 cases, the rank orders of metabolite identifications did not increase from being the only metabolite in the NMR spectrum to being one of the ten metabolites in the mixture, which is a promising result for the identification of the real unknowns in mixtures.

While hybrid MS/NMR approaches are powerful, one of their limitation is the large sensitivity difference between NMR and MS instruments [68]. Dynamic nuclear polarization and small-volume NMR technologies are expected to continue to improve and close the sensitivity gap between NMR and MS for mass limited samples [69].

## 4. Conclusions

The metabolomics field is rapidly progressing, with strong applications in parallel with methodological advancements. Most of the recent advances in NMR and MS/NMR methods have been covered in this minireview with a specific emphasis on their contribution to targeted and untargeted metabolomics. Technologies for rapid, accurate, and automated identification, and quantitation have been improving in the last 18 months. We expect that this trend to continue. For targeted metabolomics, to further increase the automation, speed, and accuracy of absolute metabolite quantitation is critical. On the identification side, there are currently about a thousand metabolites that are present in the metabolite libraries of NMR software and web servers. This number is much smaller than the estimated several hundred thousand metabolites that are present in nature. Therefore, these libraries need to continue to expand by adding more metabolites, such as by purchasing and recording more authentic standards, by extracting data from literature, and by structure elucidation of novel metabolites, for which hybrid MS/NMR methods are clearly going to be powerful. Overall, these efforts will contribute to quantitative profiling of an ever-growing number of metabolites in application studies.
